# CsPbBr_3_–CdS heterostructure: stabilizing perovskite nanocrystals for photocatalysis†

**DOI:** 10.1039/d1sc04305f

**Published:** 2021-10-22

**Authors:** Anthony Kipkorir, Jeffrey DuBose, Junsang Cho, Prashant V. Kamat

**Affiliations:** Radiation Laboratory, University of Notre Dame Notre Dame Indiana 46556 USA; Department of Chemistry and Biochemistry, University of Notre Dame Notre Dame Indiana 46556 USA; Department of Chemical and Biomolecular Engineering, University of Notre Dame Notre Dame Indiana 46556 USA pkamat@nd.edu

## Abstract

The instability of cesium lead bromide (CsPbBr_3_) nanocrystals (NCs) in polar solvents has hampered their use in photocatalysis. We have now succeeded in synthesizing CsPbBr_3_–CdS heterostructures with improved stability and photocatalytic performance. While the CdS deposition provides solvent stability, the parent CsPbBr_3_ in the heterostructure harvests photons to generate charge carriers. This heterostructure exhibits longer emission lifetime (*τ*_ave_ = 47 ns) than pristine CsPbBr_3_ (*τ*_ave_ = 7 ns), indicating passivation of surface defects. We employed ethyl viologen (EV^2+^) as a probe molecule to elucidate excited state interactions and interfacial electron transfer of CsPbBr_3_–CdS NCs in toluene/ethanol mixed solvent. The electron transfer rate constant as obtained from transient absorption spectroscopy was 9.5 × 10^10^ s^−1^ and the quantum efficiency of ethyl viologen reduction (*Φ*_EV^+^˙_) was found to be 8.4% under visible light excitation. The Fermi level equilibration between CsPbBr_3_–CdS and EV^2+^/EV^+^˙ redox couple has allowed us to estimate the apparent conduction band energy of the heterostructure as −0.365 V *vs.* NHE. The insights into effective utilization of perovskite nanocrystals built around a quasi-type II heterostructures pave the way towards effective utilization in photocatalytic reduction and oxidation processes.

## Abstract 2

The insights into effective utilization of perovskite nanocrystals built around a CsPbBr_3_–CdS heterostructure pave the way towards their utilization in photocatalytic reduction and oxidation processes.

## Graphical abstract

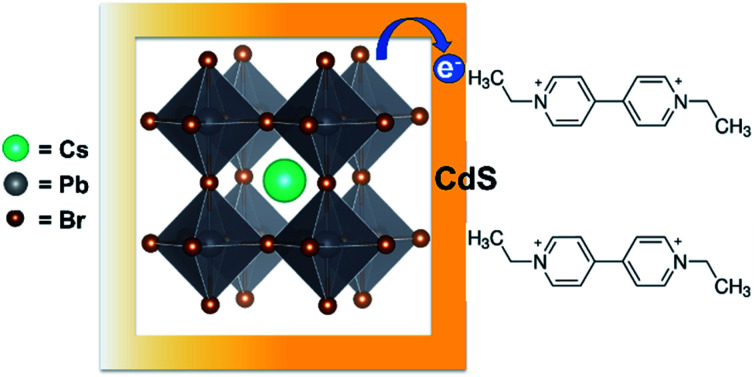

## Introduction

Semiconductor quantum dots are excellent building blocks for designing light-harvesting assemblies.^1,2^ The ability to chemically modify the surface with a functionalized ligand or to couple with another semiconductor particle offers a variety of ways to harvest visible photons.^3–5^ Since the 1990s, metal chalcogenide quantum dots (QDs), CdSe in particular, have served as the prototypical compound to elucidate excited state and charge transfer properties.^6–8^ In recent years, another quantum dot system, *viz*., perovskite nanocrystals (CsPbX_3_, X = Cl, Br, I), has emerged as a model semiconductor QD system to probe light induced optoelectronic and photocatalytic properties.^9–13^ We have recently elucidated the photocatalytic aspects of CsPbBr_3_ QDs by probing the interfacial electron transfer to methyl viologen^14,15^ and ferrocenium cation.^16^ To date, the use of these perovskite quantum dots in photocatalysis has been limited only to a few nonpolar solvents.^17–21^ The weakly-binding organic ligand shell around perovskite nanocrystals does not provide sufficient stability in polar solvents. In order to expand the scope of the perovskite nanocrystals to a wide range of photocatalytic applications (*e.g.*, solar hydrogen production or CO_2_ reduction), it is important to provide protection against chemical transformation in the presence of a redox couple or in a polar medium.

One simple approach to achieve stability in polar solvents is to cap the semiconductor nanocrystals with a thin inorganic shell. Design of such heterostructures has been successfully employed for binary and ternary semiconductors like CdSe/ZnS,^22,23^ InP/ZnS,^24^ and AgInS_2_/ZnS.^25^ The heterostructure with type I or type II band alignment offers strategies to enhance emission of the core QD or improve charge separation within the heterostructure. Although a few reports exist to-date of capping CsPbBr_3_ QDs with SiO_2_,^26^ CdS,^27^ or ZnS^28,29^ shells, none of these heterostructures have shown a major leap in achieving improved performance with a long-term stability in polar solvents. Ambiguity still exists whether the added material forms a continuous shell around the perovskite core or forms smaller discontinuous islands on the surface.^26^ Given the difficulty in imaging the thin inorganic shell around perovskite nanocrystals, because of the image contrast, one employs stability tests in a polar medium or its resistance to halide exchange to confirm surface modification.^30–33^

Designing perovskite heterostructures with metal chalcogenide shells can have several distinct advantages: (i) providing stability towards increased polarity of the solvent, (ii) remediating surface defects by directly interacting with the vacancies, and (iii) allowing for type I or quasi-type II band alignment to promote increased charge recombination in the core (increased emission yield) or improved charge separation.^34–36^ In this context, a CsPbBr_3_–CdS heterostructure offers an attractive means to tune the band energies, as their conduction bands are nearly isoenergetic (*E*_CB_ of CsPbBr_3_ and quantized CdS ≈ −0.8 V *versus* NHE)^37–39^ and facilitate charge separation. In addition, cubic CsPbBr_3_ nanocrystals have a lattice constant (*a*) of 5.85 Å (ref. 40) while that of CdS (zinc blende structure) is 5.83 Å.^41^ The similarity of the two values signifies the possibility of having a less strained interface with reduced defect states.^42^ We have now successfully prepared CsPbBr_3_–CdS heterostructures in a two-step method, and the optical properties of these structures are discussed.

## Results and discussion

### CsPbBr_3_–CdS heterostructure

**Fig. 1:**
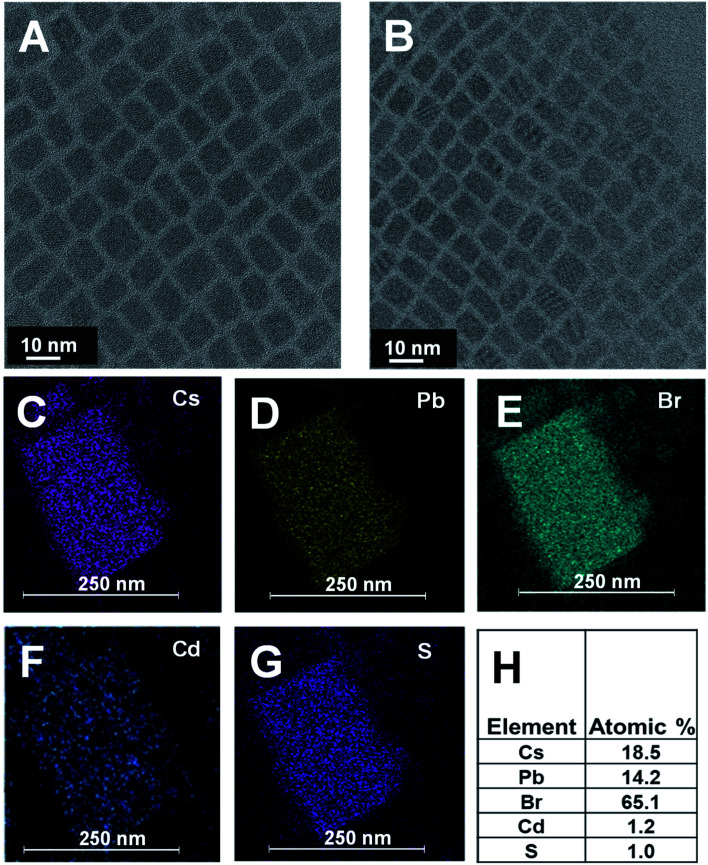
TEM image for (A) pristine CsPbBr_3_ and (B) CsPbBr_3_–CdS. (C–G) Elemental maps showing presence of Cs, Pb, Br, Cd, and S, respectively. (H) Atomic percentage of different elements as measured by EDAX.

CsPbBr_3_ quantum dots (QDs) dispersed in octadecene (ODE) were prepared using a previously reported procedure.^43^ These QDs were then treated with cadmium diethyldithiocarbamate (Cd(DDTC)_2_) at 110 °C to obtain CdS capped CsPbBr_3_ QDs. Experimental details on the synthesis of CsPbBr_3_ and CsPbBr_3_–CdS QDs are presented in the ESI (Fig. S1†). The transmission electron microscopy (TEM) images of the two nanocrystals are shown in Fig. 1A and B. These cubic particles are similar in size showing particles of lengths 8–9 nm (see Fig. S2† for size distribution analysis). This shows that CdS capping in the heterostructure is relatively thin compared to the CsPbBr_3_ core and it is difficult to identify with the image contrast in TEM images. This observation is consistent with earlier work which reports difficulty in characterizing the shell in a CsPbBr_3_ heterostructure using TEM analysis.^44,45^ These studies have attributed the imaging difficulty to low electron density contrast of the shell. However, other techniques such as elemental analysis can be useful to overcome these limitations.

We succeeded in establishing the presence of CdS in the CsPbBr_3_–CdS heterostructures through elemental analysis with TEM energy dispersive X-ray (EDX) spectroscopy. Fig. 1C–G which present elemental mapping, confirm the presence of Cd and S along with Cs, Pb, and Br. The elemental ratio (Fig. 1H) also suggests a relatively low concentration of CdS as compared to Cs, Pb and Br in the heterostructure. We can conclude that any CdS in the heterostructure is of the order of a monolayer. However, there is also the possibility of forming small clusters in and around the CsPbBr_3_ QDs.

Evidence of surface modification with CdS was also seen through the change in the surface charge. Zeta potential measurements indicated that CsPbBr_3_–CdS QDs suspended in toluene carry more negative surface charge (−37.4 mV) than pristine CsPbBr_3_ nanocrystals in toluene (−15.8 mV). This increased surface charge of CsPbBr_3_–CdS QDs enabled us to carry out electrophoretic deposition of a film under the influence of a DC field (see ESI† for details; Fig. S3†). The increased surface negativity and ability to be deposited as a film under applied bias indicates a modified surface around CsPbBr_3_. Similar electrophoretic deposition was also possible when CsPbBr_3_ nanocrystals were coated with a PbSO_4_–oleate shell.^46^ It should be noted that pristine CsPbBr_3_ QDs suspended in toluene cannot be deposited as film using electrophoresis as it does not carry sufficient surface charge.

**Fig. 2:**
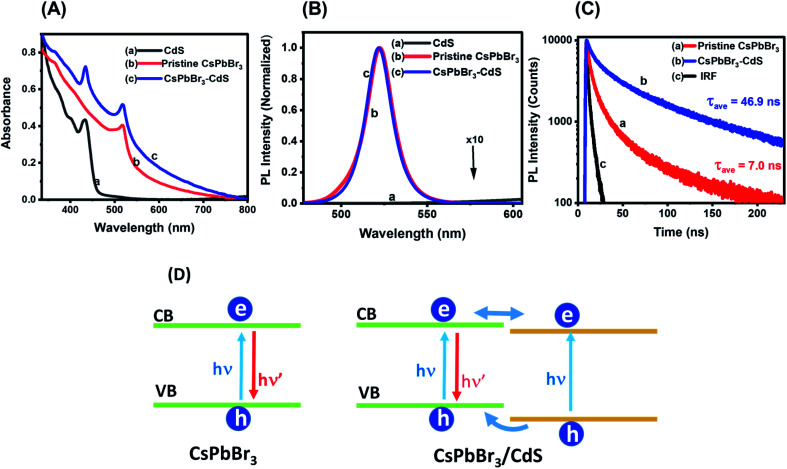
(A) Absorption spectra and (B) corresponding photoluminescence (PL) spectra of (a) CdS, (b) pristine CsPbBr_3_ and (c) CsPbBr_3_–CdS QD suspension in toluene. (C) PL decay traces monitored at 520 nm using 370 nm excitation: (a) pristine CsPbBr_3_ and (b) CsPbBr_3_–CdS nanocrystal suspensions in toluene, and (c) instrument response (IRF). The kinetic analysis is presented in the ESI.† (D) Schematic diagram illustrating charge separation and charge recombination in CsPbBr_3_ and CsPbBr_3_–CdS nanocrystals.

In addition to CsPbBr_3_ and CsPbBr_3_–CdS heterostructures, we also synthesized CdS QDs with the same ligands following a similar experimental procedure (*i.e.* without CsPbBr_3_). Fig. 2A shows the absorption spectra of CsPbBr_3_, CdS and CsPbBr_3_–CdS QDs in toluene. The CdS and CsPbBr_3_ QDs exhibit characteristic excitonic peaks at 434 and 518 nm, respectively. Although the absorption spectrum of the CsPbBr_3_–CdS heterostructure shows two peaks that overlap with the absorption of individual QDs, the peak around 434 nm may also arise from the deposition of small size CdS particles on the surface of CsPbBr_3_ nanocrystals. Such a decoration of CdS particles, if any, would give rise to a CdS excitonic peak in the heterostructures. The excitonic peak at 518 nm (CsPbBr_3_) remains unaffected after heterostructure formation, thus ruling out any interference of exchange of metal ions. Similarly, the tail absorption at longer wavelengths arises from the scattering effects, similar to what is seen in PbSO_4_–oleate capped CsPbBr_3_.^46^

The emission spectra of these three nanostructures are shown in Fig. 2B. Whereas CdS QDs remain the least emissive, both CsPbBr_3_ and CsPbBr_3_–CdS QDs are highly emissive. Additionally, the emission features of CsPbBr_3_ QDs remain unchanged following the deposition of CdS: the emission maximum (521 nm) and the full width at half maximum (18 nm) of CsPbBr_3_ are unaffected after CdS modification. These results confirm that there is no substitution of cations during the heterostructures synthesis, and thus the emission characteristics of the parent CsPbBr_3_ QDs are retained in the heterostructure. If there was any substitution of Pb^2+^ with Cd^2+^ we would expect a blue shift in the absorption and emission maxima of the perovskite QD.^47^ The excitation spectra recorded at different emission wavelengths confirm the origin of the emission to arise from the CsPbBr_3_ (Fig. S4 in the ESI†).

Another interesting aspect of the capping with CdS is the enhancement in emission yield. The emission quantum yields as determined using an integrating sphere were 39% and 60% for CsPbBr_3_ and CsPbBr_3_–CdS QDs, respectively (see ESI† for details). Passivation of surface defects by CdS is expected to suppress nonradiative processes and thus lead to increased emission yield. For example, capping of CdSe with CdS has resulted in the significant enhancement of emission yield.^48–51^ The flow of charge carriers from the CdS shell to CdSe core in these studies was established through emission and excitation spectral measurements. Control experiments were carried out to check whether introduction of Cd^2+^ ions alone can induce similar changes in the emission properties. Fig. S5† shows a decrease in emission yield and lifetime when CsPbBr_3_ was treated with cadmium acetylacetonate (Cd(acac)_2_) instead of Cd(DDTC)_2_. Additionally, treatment with Cd^2+^ did not change the absorption of the QDs. This further confirms that the observed optical properties are due to the presence of CdS in the heterostructure.

We employed time-resolved emission measurements to monitor the excited state behavior of CsPbBr_3_ before and after CdS deposition. The emission decay at 520 nm was monitored for CsPbBr_3_ and CsPbBr_3_–CdS samples (Fig. 2C). Each trace was analyzed using a biexponential decay fit and the fitting parameters are presented in Table S1.† Of interest is the increase in emission lifetime of CsPbBr_3_ upon capping with CdS. A nearly seven-fold increase in average lifetime of CsPbBr_3_–CdS QDs (*τ*_ave_ = 46.9 ns) was observed over that of pristine CsPbBr_3_ QDs (*τ*_ave_ = 7.0 ns). This shows that CdS deposition facilitates long-lived charge separation in CsPbBr_3_–CdS. In addition to surface passivation, we can also expect the formation of a quasi-type II heterojunction as shown in the scheme (Fig. 2D). Whereas direct charge carrier recombination is dominant in pristine CsPbBr_3_, the nearly isoenergetic conduction bands of CsPbBr_3_ and CdS can facilitate delocalization of electrons across the two semiconductors, thus improving charge separation. Since the CdS layer is relatively thin, its contribution to the emission is expected to be small. The excitation spectra (Fig. S4†) rule out the contribution from CdS to overall emission. The observed increase in lifetime parallels the emission yield enhancement seen in the CsPbBr_3_–CdS heterostructure.

### Stability in polar environment

Attaining long term stability of CsPbBr_3_ nanocrystals in polar solvents remains a challenge. CsPbBr_3_ nanocrystals undergo rapid degradation in polar solvents, which has hampered their applications in photocatalysis. Even the addition of a small amount of polar solvents such as ethanol or water can induce chemical transformation/precipitation of CsPbBr_3_ QDs and thus a loss of photoactivity.^9,52,53^ Recently, it was reported that ZnS-capped CsPbBr_3_ QDs were stable in a toluene : water biphasic mixture. The contact with water was made by periodic shaking since the two solvents are immiscible.^28^ We also conducted a similar stability test of CsPbBr_3_ and CsPbBr_3_–CdS nanocrystals using a biphasic mixture of toluene and water with periodic shaking. The emission spectra of CsPbBr_3_ and CsPbBr_3_–CdS nanocrystals and the PL intensity variation recorded during 30 hour period are shown in Fig. S6.† CsPbBr_3_ nanocrystals became non emissive after 20 hours of exposure in biphasic mixture. On the other hand, CsPbBr_3_–CdS nanocrystals, after an initial drop in photoluminescence, maintained more than 40% emission even after 30 hours.

**Fig. 3:**
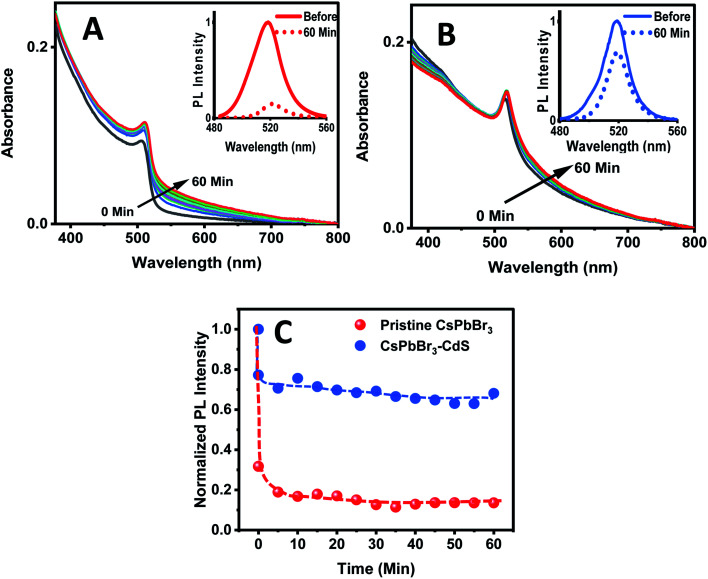
Absorption spectra of (A) CsPbBr_3_ and (B) CsPbBr_3_–CdS QDs in toluene recorded at different time intervals after addition of ethanol (15% v/v). Spectra were recorded at a time interval of 5 min. Insets: PL spectra of QDs before and 60 min after ethanol addition (excitation: 370 nm). (C) Changes in the normalized PL intensity of the CsPbBr_3_ and CsPbBr_3_–CdS QDs monitored at 520 nm as a function of time after addition of ethanol (15% v/v) to the toluene suspension.

The biphasic solvent mixture approach does not represent an increase in the overall polarity of the medium. Ideally, an inorganic shell should prevent direct contact of CsPbBr_3_ with a polar environment and maintain its photostability. We checked the stability of CsPbBr_3_ and CsPbBr_3_–CdS by introducing a miscible polar solvent (ethanol) to a toluene suspension of the QDs and monitoring the absorption and emission spectra over time. Fig. 3A and B show the absorption and emission spectra recorded following addition of ethanol (15% v/v) to toluene solution over a period of 60 minutes. The absorption of pristine CsPbBr_3_ QDs shows enhanced absorbance with time due to scattering effects caused by turbidity as the ligands from QD surface become detached in the polar medium.^54^ A ∼85% decrease in the CsPbBr_3_ emission yield is seen immediately after the addition of ethanol to the toluene solution. In addition, upon exposure to ethanol we also see a change in the absorption of CsPbBr_3_ due to particle aggregation. These results confirm the susceptibility of CsPbBr_3_ QDs to polar environment (Fig. 3C). On the other hand, CsPbBr_3_–CdS QDs exhibit only a small decrease (∼15%) in emission with a relatively small change in the absorption during 60 min of exposure in toluene/ethanol mixed solvent. The CdS deposition provides the necessary protection for CsPbBr_3_, and thus decreases its susceptibility to ethanol-induced degradation.

### Excited state interactions with an electron acceptor

**Fig. 4:**
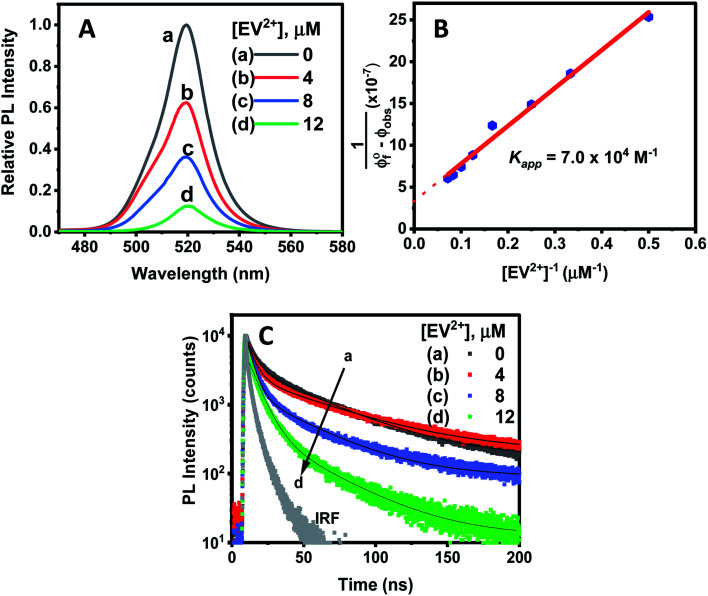
(A) PL quenching of CsPbBr_3_–CdS QDs (≈16 nM) in toluene : ethanol (90 : 10% v/v) upon additions of increasing concentration of ethyl viologen (EV^2+^) dissolved in ethanol. (B) Double reciprocal plot analysis of emission quenching of CsPbBr_3_–CdS in toluene : ethanol. The slope (3.3 × 10^−7^) and intercept (4.3 × 10^−12^ M^−1^) was used to determine the apparent association constant, *K*_app_, 7.0 (±0.8) × 10^4^ M^−1^. (C) Photoluminescence decay at different EV^2+^ concentrations, recorded with 370 nm excitation and monitored at *λ*_max_ = 520 nm.

Since CsPbBr_3_–CdS QDs were stable in toluene/ethanol mixed solvent, we were able to probe the excited state interactions and interfacial electron transfer with a cationic electron acceptor, ethyl viologen, EV^2+^. Emission spectra recorded at different concentrations of EV^2+^ are shown in Fig. 4A. The quenching of photoluminescence confirmed the excited state interaction between CsPbBr_3_–CdS QDs and EV^2+^. As a control, we tested the solubility of EV^2+^ in the toluene/ethanol mixed solvent separately by recording absorption spectra and confirming the probe molecules are fully soluble at the concentrations employed in this study (Fig. S7†). Earlier studies have shown direct complexation between CsPbBr_3_ and methyl viologen and elucidated the role of surface bound ligands in dictating the complexation constant.^14,15^ Here, we were able to quench the emission at micromolar concentrations of EV^2+^, thus indicating a complex formation in the ground state between CsPbBr_3_–CdS and EV^2+^.^51^ The equilibrium of the bound and unbound EV^2+^ molecules (reaction (1)) can be expressed in terms of the apparent association constant, *K*_app_ and emission yields (expression (2)).^55^ The observed quantum yield (*ϕ*_f_(obs)) takes into account the emission arising from EV^2+^-bound (*ϕ*_f_′) and pristine (*ϕ*_f_^0^) CsPbBr_3_ QDs. With increasing concentration of EV^2+^, more CsPbBr_3_–CdS QDs bind to viologen and thus exhibit a decrease in the emission yield.1
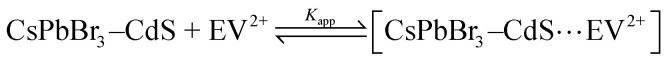
2
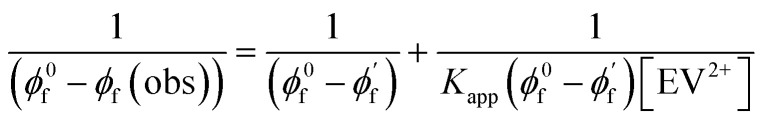


The photoluminescence quenching data was analyzed using expression (2). The emission intensity of the QDs at the emission maximum (which is proportional to quantum yield, (*ϕ*_f_(obs))) was monitored at different concentration of EV^2+^.^55,56^ The linear dependence of the double reciprocal plot (1/(*ϕ*^0^_f_-*ϕ*_f_(obs)) *versus* 1/[EV^2+^]) in Fig. 4B confirms the validity of the association between CsPbBr_3_–CdS and EV^2+^. The apparent association constant *K*_app_ determined from the slope and intercept of the plot in Fig. 4B was 7.0 × 10^4^ M^−1^. This complexation constant is 1–2 orders of magnitude smaller than the one observed for uncapped CsPbBr_3_ and viologen (0.8–7.0 × 10^6^ M^−1^).^15^ The decrease in *K*_app_ further indicates that the presence of CdS reduces the surface interactions with the viologen. The *K*_app_ value we obtain in this study is in line with literature values of CdS interacting with viologens.^57^

To further establish the excited state interactions, we monitored the photoluminescence lifetime of the CsPbBr_3_–CdS QDs at different EV^2+^ concentrations. Time-resolved luminescence decay traces were recorded in toluene : ethanol (85 : 15% v/v) using an excitation source at 370 nm. The lifetimes were fitted to a biexponential kinetic expression (expression 3), and the fitting parameters are given in Table S2.†3
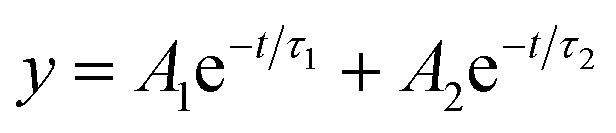


The average lifetime (*τ*_ave_) decreased with increasing concentration of EV^2+^ in accordance with the photoluminescence quenching seen in Fig. 4A. The decrease in average lifetime from 42.4 ns to 18.1 ns upon addition of 12 μM EV^2+^ is indicative of a competing excited state deactivation pathway involving electron transfer from the CsPbBr_3_–CdS QDs to EV^2+^. Since this electron transfer is likely to occur within the time resolution (∼1 ns) of our photoluminescence lifetime set up, we employed femtosecond transient absorption spectroscopy to resolve the electron transfer process.

**Fig. 5:**
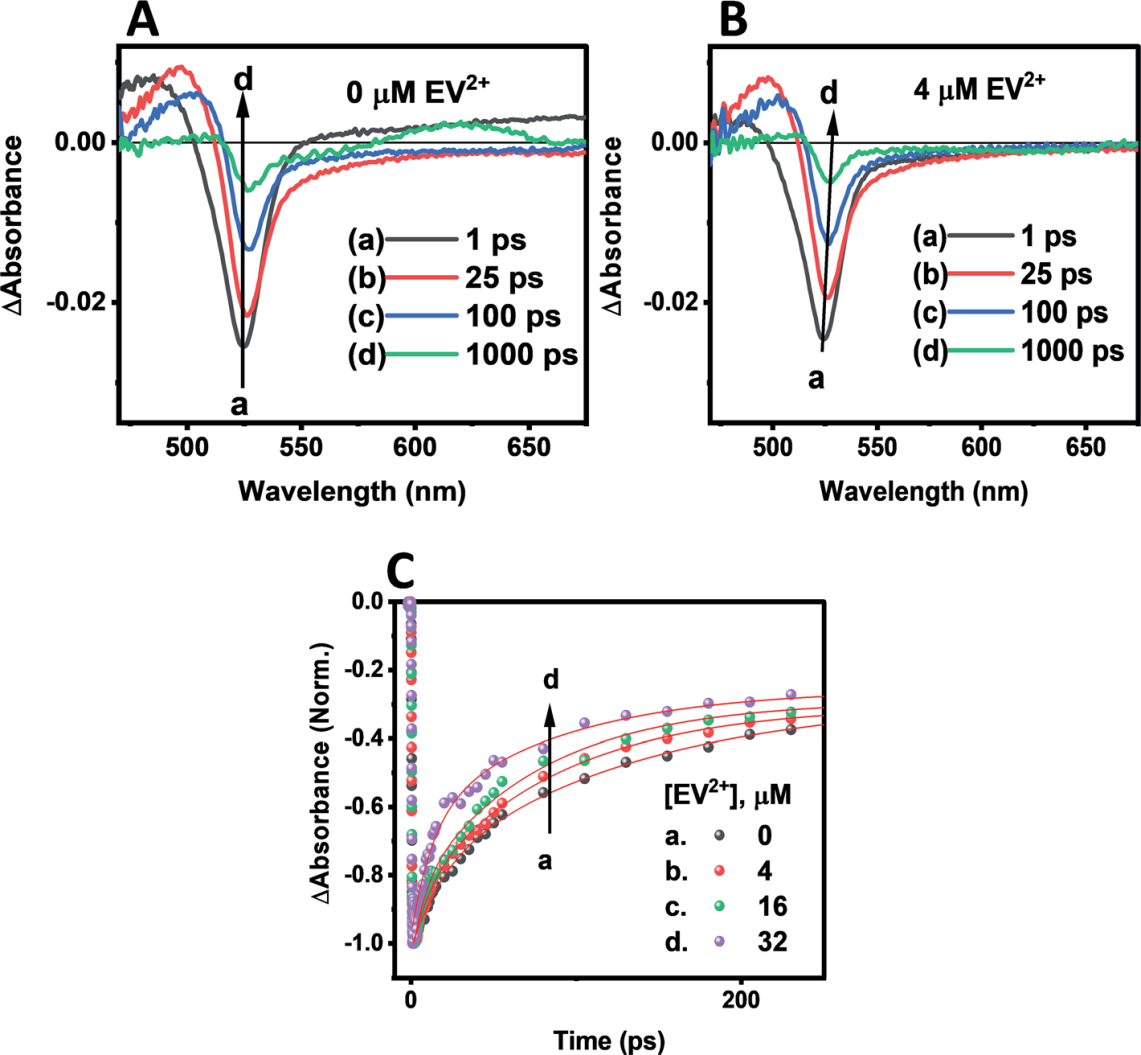
Transient absorption spectra of CsPbBr_3_–CdS suspension in toluene (≈16 nM): (A) without EV^2+^ and (B) with 4 μM EV^2+^. The difference absorption spectra were recorded following a 400 nm laser pulse (16 μJ cm^−2^) excitation. (C) Kinetic traces of the CsPbBr_3_–CdS bleach recovery at 527 nm in the absence and increasing concentrations of EV^2+^.

Transient absorption spectra were recorded following laser pulse excitation at 400 nm (16 μJ cm^−2^). The transient absorption spectra of a representative CsPbBr_3_–CdS QD sample containing 0 and 4 μM EV^2+^ are shown in Fig. 5A and B respectively. The negative absorption (exciton bleach) feature centered at ∼527 nm corresponds to the charge separated state within the NCs.^58–60^ The charge separation which occurs within the laser pulse is seen in spectrum ‘*a’* recorded with a probe delay of 1 ps. As electrons and holes recombine, a recovery in the bleached absorption is seen. The bleach recovery at 527 nm for the samples containing different amounts of EV^2+^ are presented in Fig. 5C (see Fig. S8† for longer time scale kinetics). With increasing EV^2+^ concentration we observe a quick recovery of the bleach feature, thus confirming the presence of an additional deactivation pathway for the photogenerated electrons, *viz.*, electron transfer to EV^2+^ (reaction (4)).

4 [CsPbBr_3_–CdS⋯EV^2+^] + *hν* → [(CsPbBr_3_–CdS)*⋯EV^2+^] − *k*_et_ → [CsPbBr_3_–CdS (*h*)⋯EV^+^˙]

The bleach recovery was analyzed using a biexponential kinetic fit^35^ and the fitting parameters (*a*_1_, *τ*_1_) and (*a*_2_, *τ*_2_) corresponding to fast and slow components are presented in Table S3.† While the fast component varied in the range 18.6–6.6 ps the long component varied in the range of 129.6–71.7 ps.

If we assign the decrease in the fast component to the electron transfer pathway from CsPbBr_3_–CdS to EV^2+^, we can obtain the rate constant for electron transfer through the expression (5).5
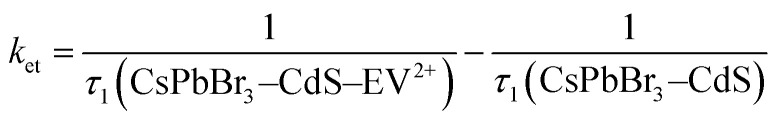


If we substitute the fast time components (*τ*_1_) of CsPbBr_3_–CdS and that of CsPbBr_3_–CdS with 32 μM EV^2+^ in expression 5 (Table S3†), we obtain rate constant (*k*_et_) of the electron transfer in the range of 3.8–9.8 × 10^10^ s^−1^ for three different EV^2+^ concentrations (4–32 μM) or an average rate constant of 6.5 × 10^10^ s^−1^. This rate constant for electron transfer is lower than the one obtained for electron transfer between oleic acid/oleylamine capped CsPbBr_3_ QDs and viologen (*k*_et_ = 3.6 × 10^11^ s^−1^).^15^ As discussed in the emission quenching experiments, the surface interactions play a role in dictating the kinetics of interfacial electron transfer.

**Scheme 1:**
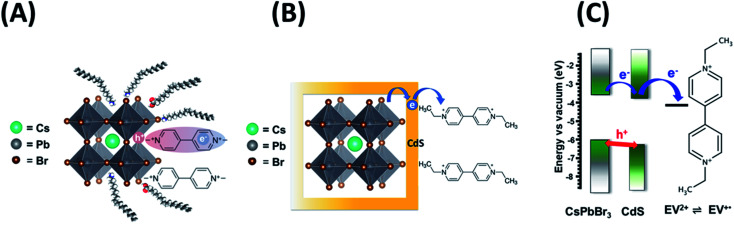
Photoinduced electron transfer between CsPbBr_3_ and viologen (A) without and (B) with mediation through a CdS layer. (C) Energetic diagram showing the flow of charge carriers and CdS mediated reduction of viologen.

It is evident that modification of CsPbBr_3_ with CdS slows down the electron transfer rate. Schemes 1A and B illustrate the two scenarios for achieving electron transfer, *viz.*, without and with CdS modification. As shown earlier, when methyl viologen is directly bound to CsPbBr_3_, the charge separation is extended, with electrons residing in the viologen moiety and holes residing within the CsPbBr_3_ QDs.^14^ This extended charge separation as a bound pair was manifested as a long-lived transient bleach component. In contrast, the electron transfer with CsPbBr_3_–CdS is mediated through the CdS layer as expected by the quasi-type II band alignment (Scheme 1C). The reduced ethyl viologen (EV^+^˙) is no longer directly bound to the CsPbBr_3_, but instead is now linked to the CdS layer. Similar CdS-mediated electron transfer has been observed in CdSe–CdS heterostructures.^51^ The bleaching recovery accelerates with increasing viologen concentrations as the electrons are depleted from the CsPbBr_3_ core, mediated through CdS. The distinct difference between the two bleach recovery kinetics observed with pristine CsPbBr_3_ and CsPbBr_3_–CdS QDs further highlights the electron mediation of CdS in promoting interfacial electron transfer in the heterostructure (Scheme 1C).

### Steady state photolysis and Fermi level equilibration

Although there have been several studies that demonstrate the photocatalytic properties through product identification,^17,18,36,61^ direct spectroscopic identification of intermediates or electron transfer products is rather limited. If indeed the CsPbBr_3_–CdS heterostructure is responsible for photocatalytic reduction, we should be able to observe the buildup of stable viologen radical under continuous photoirradiation.^62,63^

**Fig. 6:**
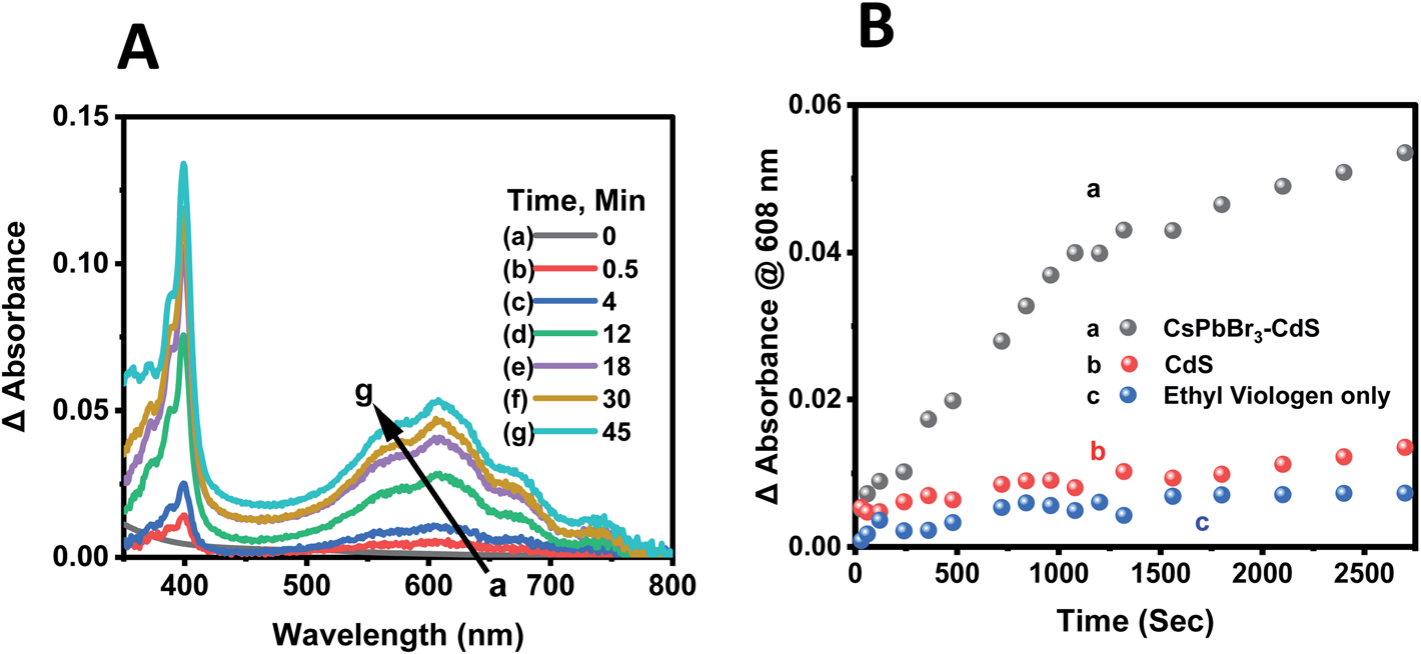
(A) Difference absorbance spectra recorded during steady state photolysis corresponding to EV^+^˙ formation. The [CsPbBr_3_–CdS + EV^2+^] sample before excitation was used as a reference. Absorption spectra were recorded at different light exposure times. (B) Evolution of EV^+^˙ formation monitored *via* the growth of absorbance at 608 nm with CsPbBr_3_–CdS. Control experiments with CdS photocatalyst and in the absence of any photocatalyst are also shown. The initial concentration of EV^2+^ was 100 μM. Experiments were carried out in deaerated toluene/ethanol 85 : 15 v/v% with visible light excitation (Xe lamp, 400 nm long pass filter, 200 mW cm^−2^).

Deaerated CsPbBr_3_–CdS nanocrystal suspensions in toluene : ethanol (85 : 15 v/v%) mixed solvent containing 100 μM of EV^2+^ were subjected to steady-state visible light illumination (>400 nm; 200 mW cm^−2^). Absorbance spectra were recorded periodically during the steady state photolysis experiment. Fig. 6A shows a representative difference absorbance spectrum, which shows distinct peaks at 405 nm and 608 nm, corresponding to the absorbance of EV^+^˙.^64^Fig. 6B shows the growth of the 608 nm absorption over time, which attains a plateau after about 45 minutes. The steady state concentration of EV^+^˙ increased with increasing concentration of EV^2+^. From the extinction coefficient of EV^+^˙ at 608 nm (*ε* = 1.4 × 10^4^ M^−1^ cm^−1^)^63,64^ we can determine the steady state concentration of the electron transfer product. The quantum yield (QY) of EV^+^˙ formation (*Φ*_EV^+^˙_) was determined using potassium ferrioxalate actinometry.^51,65^ Details of the actinometry experiments are presented in the ESI.† In the present case we obtain a maximum quantum efficiency (*Φ*_EV^+^˙_) of 8.4% for the electron transfer. Although the initial electron transfer yield as monitored from the transient absorption could be as high as 73%,^15^ the back-electron transfer during the steady state irradiation makes the net electron transfer yield lower than the value obtained immediately after laser pulse excitation.

The steady state concentration of the reduction product EV^+^˙ is dictated by the forward and back electron transfer processes. Because of the use of ethanol as a hole scavenger, the back electron transfer rate constant (*k*_bet_) is significantly lower in the present experiments. The redox couple in contact with a semiconductor surface undergoes Fermi level equilibration that is dependent on the position of the conduction band and the potential of the redox couple. The equilibrium concentration of the reduced and oxidized species can be used to determine the flat band potential of the semiconductor. The steady state concentration of [EV+˙]_ss_ is indicative of charge equilibration between the CsPbBr_3_–CdS nanocrystals and EV^2+^/EV^+^˙ couple. We employed the Nernst equation (expression (6)) to obtain the flat band potential of the CsPbBr_3_–CdS heterostructure.6
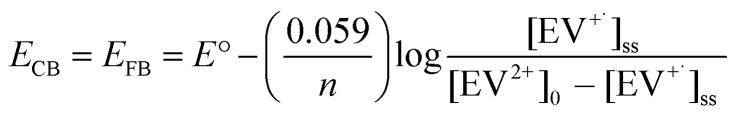


By substituting the redox potential of the EV^2+^/EV^+^˙ couple, *E*° = −0.449 V *vs.* NHE,^64^ and the steady state concentration values of the EV^+^˙ (3.65 μM) and EV^2+^ ([EV^2+^] = [EV^2+^]_0_ − [EV^+^˙]_ss_ = 96.35 μM), we obtain *E*_FB_ = −0.365 V *vs.* NHE. It is evident that the close lying conduction band potential of the CsPbBr_3_–CdS heterostructure limits the one electron transfer to EV^2+^ (Scheme 1C). It should be noted that the flat band potential of the CsPbBr_3_–CdS heterostructure obtained in this study is based on the charge equilibration between the semiconductor QD and redox couple and may differ from the values obtained from theoretical estimates or bulk material. The estimate of flat band potential provides an estimate of the energetics of the CsPbBr_3_–CdS heterostructure suspended in the solvent medium to execute photocatalytic processes.

## Conclusions

The design of CsPbBr_3_–CdS heterostructure offers stabilization of perovskite nanocrystals for photocatalytic applications in polar medium. The salient feature of the CsPbBr_3_–CdS heterostructure is realized through its stability in mixed solvents, remediation of surface defects, and increased emission yield. The stability of the CsPbBr_3_ structure in mixed solvents with increased polarity has allowed us to accumulate electron transfer product (reduced viologen) under steady state irradiation conditions with a quantum efficiency of 8.4%. The relatively high electron transfer efficiency observed in the present study shows how a heterostructure design of perovskite nanocrystals plays a crucial role in dictating the photocatalytic properties of stable perovskite nanocrystals.

## Data availability

The data is available within the main text and ESI.†

## Author contributions

AK: conceptualization, synthesis, measurements, data analysis, validation, visualization, writing first draft and revision. JTD: data analysis, discussions, revision. JC: conceptualization of material synthesis, preliminary investigation; revision. PVK: conceptualization, method development, funding acquisition, resources, supervision, discussions, writing.

## Conflicts of interest

There are no conflicts to declare.

## Supplementary Material

SC-012-D1SC04305F-s001
