# Supplementary material for: CsPbBr3–CdS heterostructure: stabilizing perovskite nanocrystals for photocatalysis
Source: Chem Sci. 2021 Oct 22;12(44):14815–25. doi: 10.1039/d1sc04305f (PMC8597851; doi:10.1039/d1sc04305f)
Supplement: SC-012-D1SC04305F-s001 [file SC-012-D1SC04305F-s001.pdf]

Electronic Supplementary Material (ESI) for Chemical Science.
This journal is © The Royal Society of Chemistry 2021

Supporting information

$CsPbBr_3$–CdS Heterostructure: Stabilizing Perovskite Nanocrystals for Photocatalysis

Anthony Kipkorir,[1,2] Jeffrey DuBose,[1,2] Junsang Cho[1] and Prashant V. Kamat[1,2,3*]

[1]Radiation Laboratory, [2]Department of Chemistry and Biochemistry, and

[3]Department of Chemical and Biomolecular Engineering

University of Notre Dame

Notre Dame, Indiana 46556, United States

*Corresponding Author: pkamat@nd.edu

Chemicals

Cesium carbonate (Cs_2CO_3, 99.9%, Sigma-Aldrich), lead (II) bromide ($PbBr_2$, 99.999%, Sigma-Aldrich), oleic acid (OA, 90%, Sigma-Aldrich), oleyl amine (OAm, technical grade 70%, Sigma-Aldrich), 1-octadecene (ODE, technical grade 90%, Sigma-Aldrich), Cadmium nitrate ($Cd(NO)_3$.4H2O, Sigma-Aldrich), sodium diethyldithiocarbamate ($Na(DDTC)_2$, Flinn Scientific), cadmium acetylacetonate ($Cd(acac)_2$), ≥99.9% trace metals basis, Sigma Aldrich), hydrobromic acid (HBr, 48% w/w in H_2O, Sigma-Aldrich), methyl acetate (Me-OAc, 99.98%, ethyl viologen dibromide (Sigma-Aldrich), toluene (Anhydrous, 99.99%, Sigma-Aldrich, ethanol (200 proof, Koptec), acetonitrile (99.9%, Sigma-Aldrich).

Synthesis of $Cd(DDTC)_2$

The $Cd(DDTC)_2$ was prepared using a modified form of the method reported by Youn et al. [1] . 1 mmol (308 mg) of $Cd(NO)_3$ in 20 ml ethanol and 2 mmol (451 mg) of Na(DDTC) in 20 ml ethanol in a 50 mL beaker each. The two were then transferred to a 250mL beaker and stirred at room temperatures for 1 hour. The white precipitate formed ($Cd(DDTC)_2$) was then Vacuum filtered through a (Whatman) filter paper. Deionized water was used to wash the product and was thereafter dried in an oven at 80°C under vacuum.

Synthesis of $CsPbBr_3$ nanocrystals (NCs)

$CsPbBr_3$ NCs were synthesized following a modified procedure adapted from Protesescu *et al.* [2].

Cs-Oleate was prepared by mixing 153 mg of Cs_2CO_3, 1.4 mL of Oleic Acid, and 3 mL of 1-Octadecene in a 25 mL round bottom flask. The mixture was degassed at 80˚ C for 1 hour after which the temperature was raised to 120˚C and maintained here under nitrogen until injection time.

The lead- bromide precursor was prepared by adding 3 mL of Oleic Acid, 3 mL of 1-Octadecene, and 414 mg of $PbBr_2$ to a 25 mL round bottom flask and degassing for 1 hour at 80˚ C. After one hour, the temperature was raised to 170˚ C and 3 mL of oleylamine were added to the mixture. Degas it for about a minute more. The vacuum was then turned off and the flask was put into a nitrogen environment.

When the said temperatures of both solutions are obtained, 2 mL of Cs-Oleate were injected into the lead-bromide precursor with a heated syringe (from the oven). Within the 5 seconds of injection, the mixture was put in an ice bath until it reached a temperature of 65˚ C. The nanocrystals were washed with approximately 20 mL of 1-Octadecene and transferred to a 50mL centrifuge tube. This was then spun down for 10 minutes at 7000 rpms. The precipitate/pellet was kept and dispersed in 5mL toluene washed with 5 mL of methyl acetate. The nanocrystals were spun down again for 10 minutes at 700 rpms. This last step of dispersing in toluene and washing with methyl acetate was repeated three times. The resulting pellet was air dried and kept for the next synthetic steps.

Synthesis of $CsPbBr_3$-CdS core/shell heterostructure

$CsPbBr_3$-CdS core/shell QDs was synthesized following a modified procedure of Ravi *et al*[3]. 15 mg $CsPbBr_3$ QDs powder, 20 µl of OAmBr and 3ml of ODE are mixed and sonicated for 3 minutes before adding 5 mg of $Cd(DDTC)_2$ powder and sonicating it further for 5minutes. The reaction mixture is degassed at room temperature for 10 minutes then heated to 110 °C where the temperature is maintained for 1 hr. The crude solution is cooled to room temperature and centrifuged at 7000 rpm for 10 minutes. The resulting precipitate is then dispersed in 4mL toluene and 4 mL MeOAc is added as antisolvent; this is then centrifuged at 7000 rpm for 10 minutes. This last washing step is repeated 2 times. The nanocrystals are then dispersed in toluene.

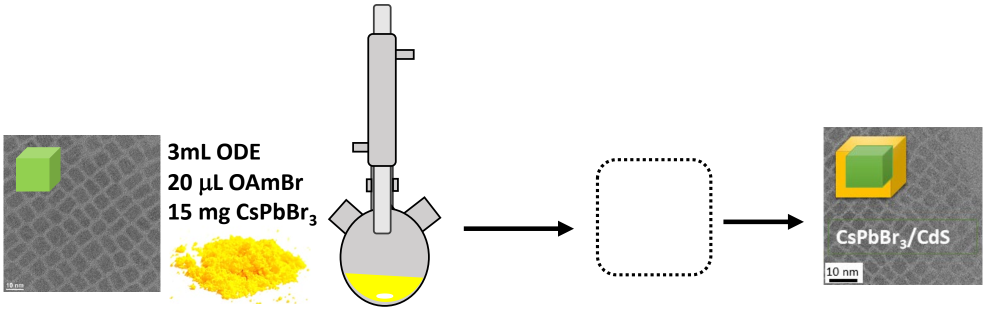

Element	Cs	Pb	Br	Cd	S
Atomic %	18.5	14.2	65.1	1.2	1.0

Figure S1. **(A)** Schematic diagram of the synthesis of $CsPbBr_3$-CdS with TEM images of the pristine $CsPbBr_3$ and $CsPbBr_3$-CdS. **(B)** Atomic percentage of different elements as measured by energy dispersive X-ray spectroscopy (EDX) measurement.

Characterization

UV–visible absorption spectra were measured using a Cary 50 Bio spectrophotometer (Varian). PL spectra and absolute PL quantum yield were recorded using Ocean Optics QEPro spectrophotometer and integrating sphere respectively; both utilizing 365 nm light source. The photoluminescence decay of the samples was monitored and recorded with a time-correlated single-photon counting (TCSPC) technique on a Horiba Jobin Yvon system using IBH DataStation Hub for timing with a 371 nm nanoLED light source. Transmission electron microscopy (TEM) images and high-resolution TEM (HRTEM) images are captured by using a Titan 80-300 microscope operated at 300 kV. Energy Dispersive X-Ray (EDX) measurements are also done on S/TEM-EDX mode of the same Titan 80-300 microscope by utilizing a dedicated retractable EDX detector. The detector offers high spatial resolution hence resolving the components by

multivariate analysis before the structure is significantly changed during electron beam irradiation.

Femtosecond transient absorption measurements were carried out using a Spectra-Physics Solstice Ace laser system (800 nm fundamental, ~1 mJ/pulse, fwhm = ~30 fs, repetition rate of 1 kHz) with detection software from Ultrafast Systems (Helios). The fundamental beam was split 95/5, where 95% of the beam is frequency-doubled to 400 nm (using BBO crystal) to generate the pump beam and the remaining 5% is sent to a computerized optical delay stage and then focused onto a sapphire crystal to generate the white-light continuum probe pulse. The pump and probe beams are spatially overlapped onto the sample such that the probe is within the area of pump irradiation. The pump beam was sent through an iris of known diameter and the power was modulated via a neutral density wheel. Time-resolved absorption spectra were recorded using Argon purged toluene (or toluene/ethanol) solutions of QDs in quartz optical cells. Kinetic traces were generated from the time-resolved data sliced at different wavelengths.

Size Distribution Analysis

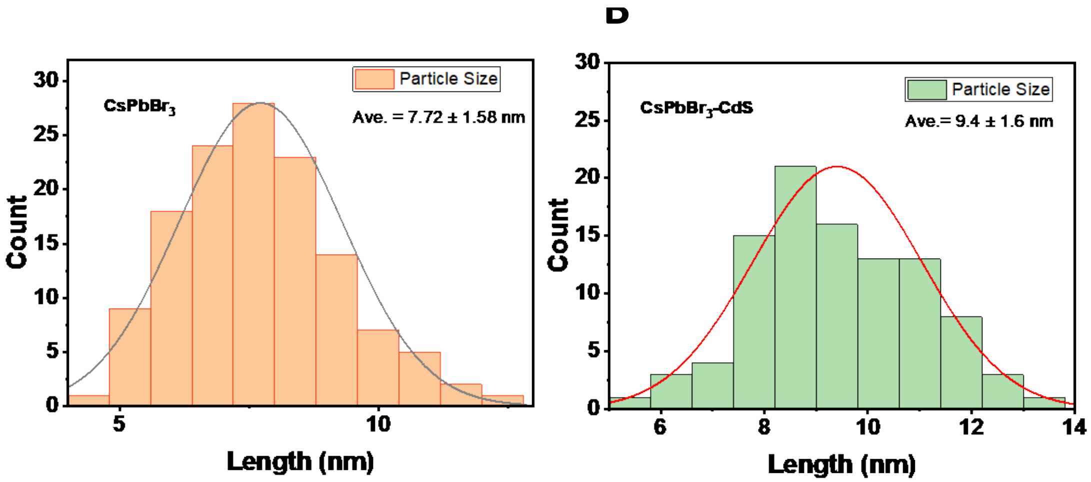

Figure S2. Size distribution statistics of (A) Pristine $CsPbBr_3$ and (B) $CsPbBr_3$-CdS. Particles were sized using ImageJ software from the TEM images in Figure 1 of the main text.

Electrophoretic deposition

Electrophoretic deposition (EPD) was done using a home-made EPD set up. A DC bias of 100 V was applied across a ~1 cm gap between an FTO counter electrode and an FTO/TiO_2 film (working electrode). The $CsPbBr_3$-CdS nanocrystals suspended in a toluene solution respond to the applied field and deposit onto the TiO_2 surface. The loading of the film could be monitored by recording the decrease in the absorbance of the deposition solution, or by recording the absorbance of the FTO/TiO_2/$CsPbBr_3$-CdS film itself (Figure S3A).

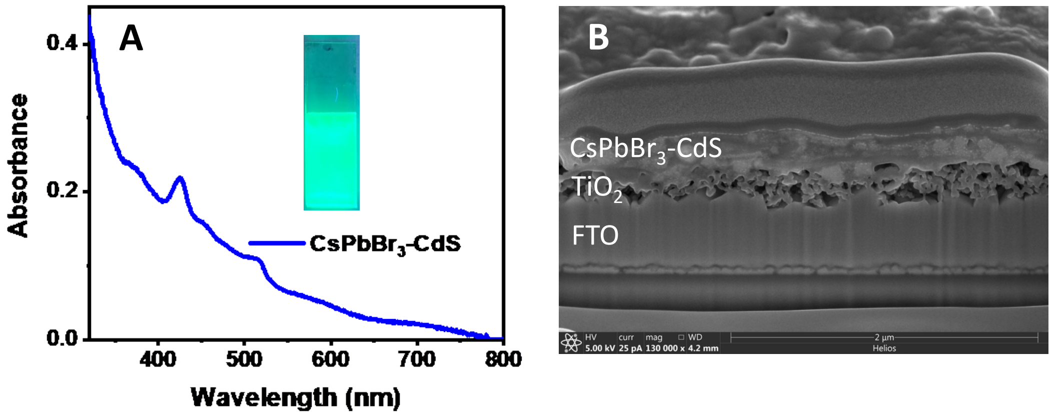

Figure S3: (A) Absorption spectra of the EPD-deposited $CsPbBr_3$-CdS film (Inset: $CsPbBr_3$-CdS film under UV light). (B) Cross-sectional SEM image of $CsPbBr_3$-CdS deposited on mesoscopic TiO_2 film using electrophoretic deposition.

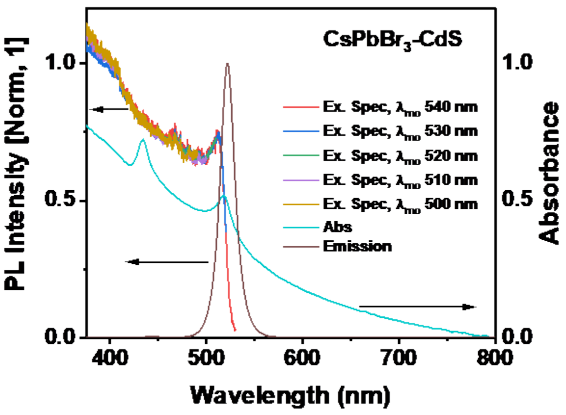

Figure S4: Excitation spectra $CsPbBr_3$-CdS NCs in toluene (deaerated) recorded at different monitoring wavelength (λ_{mo}) shows no contribution to emissions from longer wavelength region. Absorption and emission spectra of the corresponding sample are included for comparison.

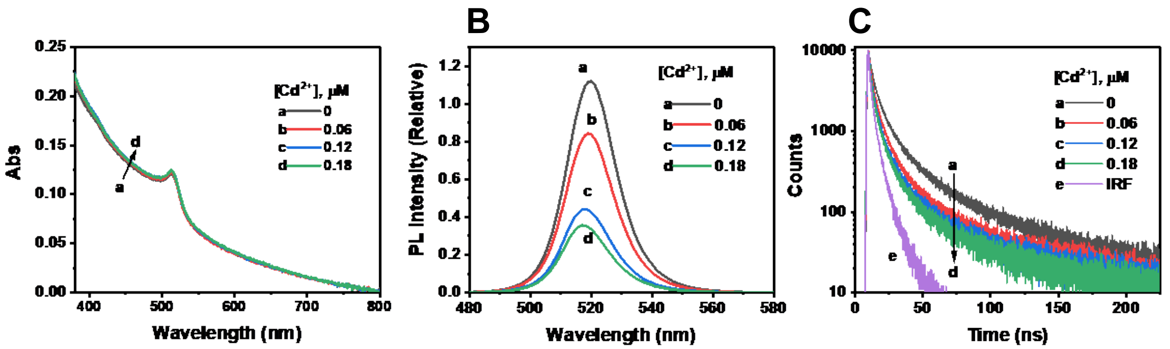

Figure S5: (A) Absorption, (B) emission spectra, and (C) emission decay traces of $CsPbBr_3$ QDs recorded with following treatment with 0 - 0.18 µM $Cd(acac)_2$. $CsPbBr_3$ QDs were dispersed in toluene, and all measurements were done under deaerated conditions. Excitation wavelength for the emission and emission decay measurements was 370 nm.

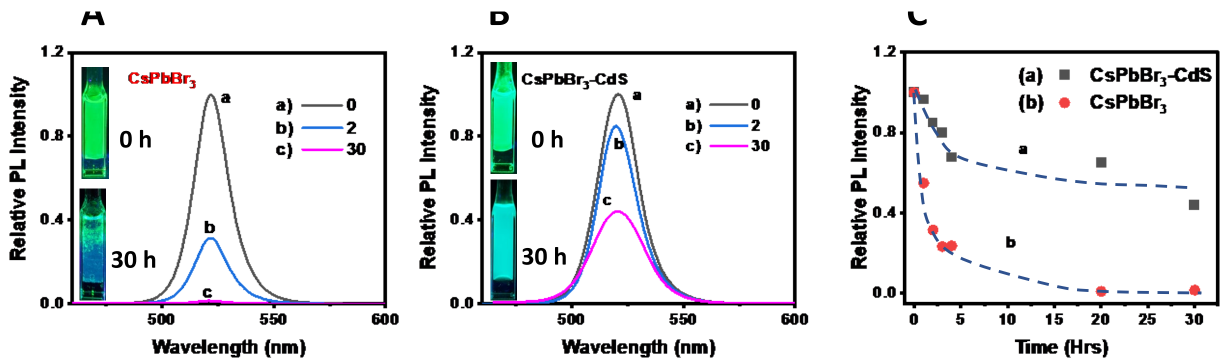

Figure S6. Stability test with periodic exposure of toluene suspension to water: (A) pristine $CsPbBr_3$ and (B) $CsPbBr_3$-CdS NCs. The photograph of photoluminescence of the two nanocrystals in biphasic mixture of toluene and water at time 0 and 30 hours are shown in the inset. (C) The changes in the PL intensity with time of $CsPbBr_3$ and $CsPbBr_3$ –CdS in a biphasic solvent mixture. After ~20 hrs. the pristine $CsPbBr_3$ shows complete PL quenching while the $CsPbBr_3$ -CdS retains >40% PL intensity even after 30 hours.

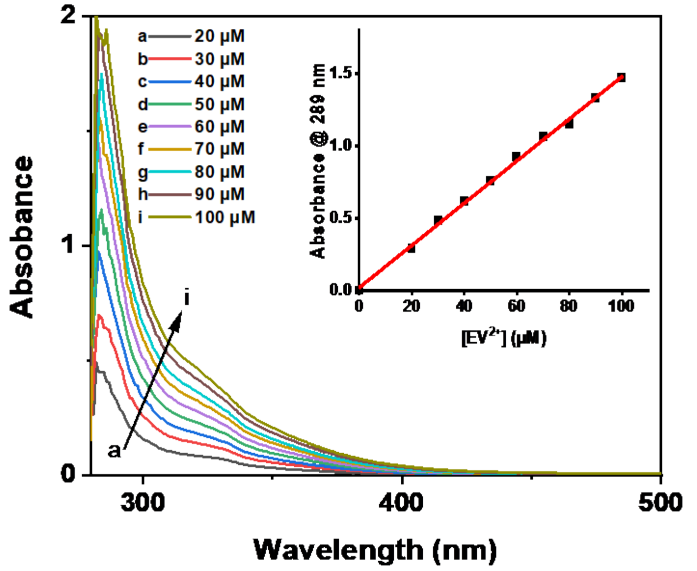

Figure S7: Evaluating solubility of ethyl viologen dibromide ($EVBr_2$) in 85:15, toluene: ethanol (v/v). Absorption spectra of EV^{2+} concentrations 20-100 µM. **Inset:** the linear relation between the peak absorbance at 289 nm *vs.* concentration. Beer-Lambert Law is obeyed (Abs = ε x concentration x path length) throughout the concentration range. 100 µM of $EVBr_2$ was adopted for use in the experiment.

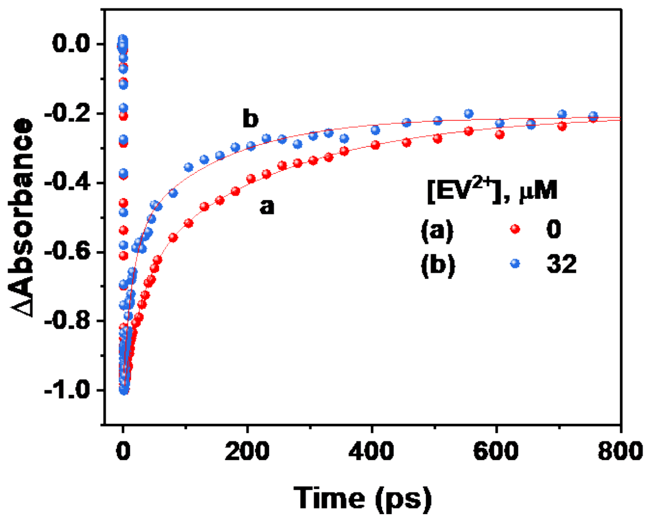

Figure S8: Kinetic traces of the $CsPbBr_3$-CdS band-edge bleach recovery at 527 nm in the absence and with 32 µM ethyl viologen. (These kinetic traces correspond to Figure 5C in the main text)

Table S1. Fitting parameters of the kinetic analysis of photoluminescence decay of pristine $CsPbBr_3$ and $CsPbBr_3$-CdS.

Sample	a_1 (%)	τ_1 (ns)	a_2 (%)	τ_2 (ns)	τ_{ave} (ns)
**Pristine $CsPbBr_3$**	95.1	3.7	4.9	18	7.0
**$CsPbBr_3$-CdS**	73.8	7.7	26.2	60.9	46.9

Table S2. Fitting parameters of the kinetic analysis of photoluminescence decay of $CsPbBr_3$-CdS with increasing concentration of EV^{2+}.

$[EV^{2+}]$, (μM)	a_1 (%)	τ_1 (ns)	a_2 (%)	τ_2 (ns)	τ_{ave} (ns)
**0**	21.7	5.63	78.3	43.67	42.35
**3**	23.3	4.75	76.7	49.24	47.98
**4**	26.7	5.31	73.3	52.90	51.22
**6**	32.8	5.32	67.2	50.27	48.06
**8**	47.2	4.63	52.8	37.36	34.10
**10**	56.9	4.52	43.1	27.81	23.69
**12**	58.0	3.66	42.0	21.46	18.07

Table S3. Fitting parameters of the kinetic analysis of bleaching recovery of $CsPbBr_3$-CdS at 527 nm with increasing concentration of EV^{2+}.

$[EV^{2+}]$, μM	a_1(%)	τ_1 (ps)	a_2(%)	τ_2 (ps)	τ_{ave} (ps)	k_{et}, ($x10^{10}s^{-1}$)
**0**	29.3	18.6	70.7	129.6	123.4	---
**4**	24.6	10.9	75.4	86.1	83.1	3.8
**8**	27.5	10.0	72.5	87.1	83.9	4.6
**16**	26.8	7.7	73.2	75.1	72.7	7.6
**32**	46.56	6.6	54.3	71.7	66.8	9.8

Fitting parameters and rate constant of electron transfer are determined using the expressions below.

$$y = A_1 e^{-t/\tau_1} + A_2 e^{-t/\tau_2}$$

Electron transfer rate constant was calculated using expression below.

$$k_{et} = \frac{1}{\tau_1(\mathrm{CsPbBr_3{-}CdS{-}}EV^{2+})} - \frac{1}{\tau_1(\mathrm{CsPbBr_3}{-}CdS)}$$

Actinometry - Quantum yield of electron transfer to EV^{2+}.

To calculate the $EV^{+\bullet}$ quantum efficiency, we adopted a modified standard potassium ferrioxalate ($K_3[Fe(C_2O_4)_3]$) actinometry[5,6]. Stock solutions of 0.2 % w/w of 1, 10-phenanthroline in water, 0.2 M ferric sulfate ($Fe_2(SO_4)_3$) in 6% H_2SO_4, sodium acetate buffer in 1% H_2SO_4, and 1.2 M potassium oxalate ($K_2C_2O_4$) in deionized water were made and stored in amber vials due to their sensitivity to light. To synthesize $K_3[Fe(C_2O_4)_3]$, 5 mL of $Fe_2(SO_4)_3$ and 5 mL of $K_2C_2O_4$ were put into a 100 mL volumetric flask and diluted to the mark with DI water. Aliquots (4 mL) of the actinometer solution were then irradiated with visible light using a 457 nm narrow bandgap filter for 14 min (corresponding to irradiation time in **Figure 6B**). 1 mL of the irradiated actinometer solution was transferred into a 10 mL volumetric flask with 2 mL of the 0.2% phenanthroline and 0.5 mL acetic acid buffer then diluted to the mark with DI water. A blank of non-irradiated actinometer solution was also replicated. The absorbance of the resulting solution was measure at 510 nm. The quantum yield of electron transfer from NRs to EV^{2+} was calculated using equation below.

$$\phi_{ET} = \frac{A_{EV^{+\bullet}}\ \varepsilon_F}{A_F\ \varepsilon_{EV^{+\bullet}}}\phi_F$$

$\phi_{F_{(457\ nm)}} = 0.85$

$A_{EV^{+\bullet}} = 0.05$

$A_F = 0.4$

$\varepsilon_{EV^{+\bullet}} = 1.40x10^4 M^{-1}cm^{-1}$

$\varepsilon_F = 1.11x10^4 M^{-1}cm^{-1}$

Φ_{ET} = 0.084 (or 8.4%)

Quantum yield measurements.

Absolute quantum yield was determined using an integrating sphere setup. Nanocrystals dispersed in toluene were put in 10 mm × 10 mm quartz cuvettes and filled to 3.5 mL. The reference spectrum of the excitation source is first taken before the sample is put into the integrating sphere. Thereafter, the emission spectra of the sample alongside that of the reference (excitation source) are taken when the cuvette is in the light path and out of path. The obtained spectra are integrated, and their areas used in calculating the PL QY as per the equation below[7].

$$\phi = \frac{P_c - (1-A)P_b}{L_a A}$$

L_a - area of light source spectrum before introducing the sample

L_b - area of light source spectrum when the sample is out of light path

L_c - area of light source spectrum when the sample is in light path

P_b - area of emission spectrum when sample is out of light path

P_c - area of emission spectrum when sample is in light path

$$A = 1 - \frac{L_c}{L_b}$$

References.

1. Youn, K.S.; Yu, K. H. Growth of CdS thin films by using MOCVD with a single-source precursor C10H20CdN2S4 or C14H28CdN2S4. *The journal of the Korean Physical Society.* **47**, 89–93 (2005).

2. Protesescu, L. *et al.* Nanocrystals of Cesium Lead Halide Perovskites (CsPbX3, X = Cl, Br, and I): Novel Optoelectronic Materials Showing Bright Emission with Wide Color Gamut. *Nano Letters* **15**, 3692–3696 (2015).

3. Kumar Ravi, V., Saikia, S., Yadav, S., v. Nawale, V. & Nag, A. CsPbBr3/ZnS Core/Shell Type Nanocrystals for Enhancing Luminescence Lifetime and Water Stability. *ACS Energy Letters* **5**, 1794–1796 (2020).

4. Michaelis, L. & Hill, E. S. THE VIOLOGEN INDICATORS. *The Journal of general physiology* **16**, 859–873 (1933).

5. Hatchard, C. G., Parker, C. A. & Bowen, E. J. A new sensitive chemical actinometer - II. Potassium ferrioxalate as a standard chemical actinometer. *Proceedings of the Royal Society of London. Series A. Mathematical and Physical Sciences* **235**, 518–536 (1956).

6. L. Bridewell, V., Alam, R., J. Karwacki, C. & v. Kamat, P. CdSe/CdS Nanorod Photocatalysts: Tuning the Interfacial Charge Transfer Process through Shell Length. *Chemistry of Materials* **27**, 5064–5071 (2015).

7. de Mello, J. C., Wittmann, H. F. & Friend, R. H. An improved experimental determination of external photoluminescence quantum efficiency. *Advanced Materials* **9**, 230–232 (1997).
